# Identification of Premature Infants at High Risk of Late Respiratory Diseases: A Retrospective Cohort Study

**DOI:** 10.3389/fped.2022.869963

**Published:** 2022-04-20

**Authors:** Ling Sun, Yingying Bao, Hong Zhang, Jiajun Zhu

**Affiliations:** Department of Neonatology, Women’s Hospital, Zhejiang University School of Medicine, Hangzhou, China

**Keywords:** late respiratory diseases, premature infants, very low birth weight, respiratory support, risk factors

## Abstract

**Aim:**

To identify infants with very low birth weight at risk of late respiratory diseases after discharge.

**Methods:**

This retrospective longitudinal study included 388 preterm infants with gestational age of < 32 weeks and birth weight of < 1,500 g and evaluated perinatal information, assessments performed while in the neonatal intensive care unit, and longitudinal follow-up *via* questionnaire until the corrected gestational age of 18–24 months.

**Results:**

The mean birth weight and gestational age were 1,191.2 ± 191.8 g and 29.1 ± 1.4 weeks, respectively. Sixty-four (16.5%) infants developed late respiratory diseases after discharge to the corrected gestational age of 18–24 months. Univariate analyses showed that gestational age, birth weight, respiratory support, oxygen use, bronchopulmonary dysplasia diagnosed at 36 weeks’ postmenstrual age and length of hospital stay were associated with late respiratory diseases. After adjusting for covariates, respiratory support was significantly associated with serious respiratory morbidities to 18–24 months corrected gestational age. With each day of respiratory support, the odds of late respiratory diseases increased by 1.033-fold.

**Conclusion:**

Respiratory support was associated with increased odds of developing late respiratory diseases during early childhood, which may be an early predictor to late respiratory morbidities. Thus, it is imperative to identify a safe and effective strategy to prevent chronic dependency on respiratory support.

## Introduction

The number of preterm births has been increasing over the past several decades (1). Early delivery in such births disrupts fetal lung development, translating to negative impacts on postnatal lung development and resulting in morbidities and reduced lung function (2). This is evidenced by the high rates of respiratory morbidities after discharge in newborns with a very low gestational age (GA) (3).

Studies have found that premature birth is associated with severe respiratory diseases, including bronchopulmonary dysplasia (BPD). Such diseases can potentially persist into adolescence and adulthood but are particularly problematic during the first 2 years of life (4). The incidence of BPD, diagnosed at 36 weeks postmenstrual age (PMA), has been used both as a predictor and a surrogate of late respiratory morbidities (5–7). However, a substantial proportion of very low birth weight (VLBW) infants with or without BPD show respiratory limitations at school age and in adulthood (8–10).

Currently, BPD is considered to be an imperfect predictor of long-term pulmonary outcomes (11). A previous study indicated that more than 50% of premature infants, including those without a formal BPD diagnosis, require subsequent rehospitalizations or chronic respiratory medications after discharge (12). Moreover, our previous study (13) found that BPD in preterm infants has a low predictive value for late respiratory morbidities. This suggests that many factors have an impact on long-term pulmonary outcomes during early childhood, leading to challenges in predicting the long-term respiratory outcomes of VLBW infants. Various studies have associated late respiratory morbidities with male sex, decreased intrauterine growth, singleton birth, exposure to young children, sociodemographic factors, and BPD (14, 15). However, the exact relationship of prenatal and early postnatal exposures with the development of late respiratory diseases (LRD) remains uncertain.

Understanding the etiology and risk factors for long-term respiratory morbidities associated with premature birth may aid in the prevention and treatment of LRD (4). Currently, data on factors that predict the likelihood of infants developing LRD are insufficient. Therefore, this longitudinal retrospective cohort study aimed to investigate potential antenatal and postnatal risk factors associated with early childhood LRD in preterm infants.

## Materials and Methods

### Study Population and Data Collection

This was a secondary analysis of the data from our previous study (13). This retrospective study was conducted using a cohort of premature infants admitted to a tertiary hospital in Hangzhou, China, from 2016 to 2018. All patients who met the following inclusion criteria were enrolled: (1) admission to the neonatal intensive care unit (NICU), (2) GA < 32 weeks, and (3) BW < 1,500 g. Our exclusion criteria described below: (1) severe congenital malformations or death during hospitalization and (2) transfer to another hospital or discharge before 34 weeks PMA. Patients lost to follow-up were excluded from the analyses. The institutional review board approved this study (IRB-20210189-R), and informed consent was obtained from the parents or guardians of the participants.

Prenatal, postnatal, and demographic information was collected by investigators. We documented prenatal factors, including maternal chorioamnionitis, preeclampsia, antenatal steroid use, and maternal education. Other variables included BW, GA, sex, mode of delivery, surfactant administration, BPD, respiratory support duration, hospital stay length, and whether or not the infant was small for their GA (BW <10th percentile). The number of LRD events limited the number of predictors in the model to seven, which were selected based on prior clinical knowledge.

Respiratory support included the use of invasive ventilation or non-invasive respiratory support that provided positive end-expiratory pressure or oxygen. This included continuous positive airway pressure, high flow (>2 L/min) nasal cannula, non-invasive intermittent positive pressure ventilation, biphasic continuous positive airway pressure, and non-invasive high-frequency oscillation.

### Outcomes

The primary outcome was LRD from discharge until follow-up at the corrected gestational age of 18–24 months. LRD were defined as any of the following (7, 16–19): (1) tracheostomy, (2) hospitalization at ≥ 50 weeks PMA for respiratory illness, (3) need oxygen or respiratory support or respiratory surveillance (e.g., apnea monitor or pulse oximeter) at following-up to 18–24 months corrected gestational age, and (4) at least two rehospitalizations due to respiratory illness from discharge to the follow-up endpoint at 18–24 months corrected gestational age. In this cohort, the 75th percentile for the number of rehospitalizations was two.

Follow-up ended in August 2020. The questionnaire was sent every 6 months, to the guardians or parents to obtain data of late respiratory outcomes. Our questionnaire comprised items involving the mode of respiratory support (e.g., invasive ventilation, non-invasive ventilation), the oxygen or respiratory surveillance (e.g., apnea monitor and pulse oximeter), frequency of airway infection, respiratory symptoms (e.g., cough or wheeze without a cold at least once a week), and hospital readmissions due to respiratory illness. We excluded infants whose guardians or parents refused to answer questions or lost to follow-up.

### Statistical Analyses

Measures of central tendency and dispersion were estimated for continuous outcome measures. Depending on their distribution, the data are presented as means and standard deviations or as medians and interquartile ranges. Frequency measures were presented as numbers (*n*) and frequencies (%). Demographic characteristics and outcomes were compared between infants with and without LRD. The differences between the two groups were determined using the independent samples *t*-test for normally distributed variables and the Mann–Whitney *U*-test for non-normally distributed variables. The chi-square test was used for categorical variables.

The number of covariates was selected based on the number of respiratory events (20). Univariate logistic analyses were performed to identify the risk factors for LRD. Baseline characteristics that were significant at *P* < 0.20 in univariate analysis were included in the multiple regression models. Considering the number of infants with LRD (*n* = 64) in our study, seven variables were chosen for multivariable analysis based on previous results and clinical constraints to avoid overfitting in the model. In cases of collinearity between variables (e.g., respiratory support and oxygen use), the medical condition was retained in the model. Odds ratios (ORs) and 95% confidence intervals (CIs) were estimated. As BW and GA are highly correlated, only BW was included in the model to estimate the LRD rates. Statistical analyses were conducted using IBM SPSS Statistics for Windows, version 26.0 (IBM Corp., Armonk, NY, United States) and GraphPad Prism 9.0 (GraphPad Software, La Jolla, California). The level of significance for the two-sided test was set at *P* ≤ 0.05.

## Results

**FIGURE 1:**
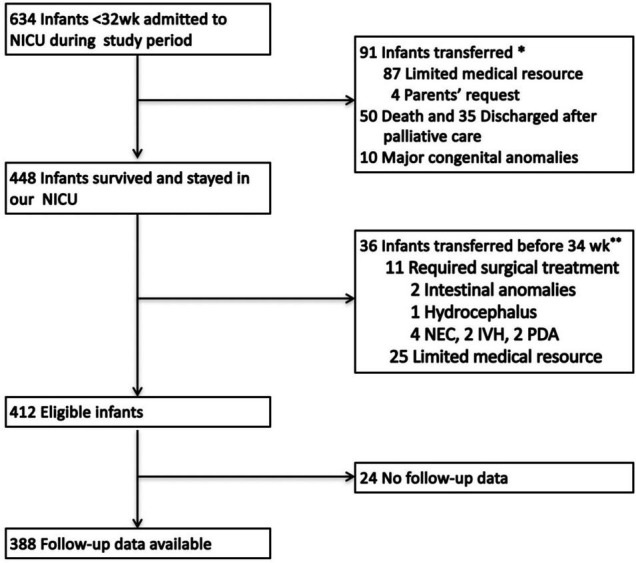
Application of inclusion and exclusion criteria to determine the number of eligible study participants. *NICU*, neonatal intensive care unit; *NEC*, necrotizing enterocolitis; *IVH*, severe intraventricular hemorrhage; *PDA*, patent ductus arteriosus. *Early transfer: Ninety-one patients were transferred early, within 48 h of birth. Among them, 87 patients were transferred to another tertiary center in our city due to limited medical resources, and four patients were transferred to other hospitals upon parents’ request. **Late transfer: Among the 36 patients transferred before 34 weeks of PMA, 25 were transferred due to limited medical resources, and 11 who required surgical treatment were transferred due to the lack of a surgery department in our hospital.

There were 412 eligible infants, among which 24 lost to follow-up, 388 patients qualified to participate in the analytic cohort (Figure 1). The characteristics of the 388 infants are shown in Table 1. The mean BW and GA were 1,191.2 ± 191.8 g and 29.1 ± 1.4 weeks, respectively, and 53.9% of the infants were male. Additionally, 10.8% of the patients were intubated immediately after birth, 88.9% of the infants received non-invasive respiratory support, and 19.1% received mechanical ventilation during hospitalization. Overall, 64 (16.5%) infants showed LRD outcomes after discharge by the corrected gestational age of 18–24 months.

**TABLE 1:** Characteristics of infants with and without late respiratory diseases.

Characteristic	All subjects (*n* = 388)	No LRD (*n* = 324)	LRD (*n* = 64)	*P*-value
Gestational age, weeks, mean (*SD*)	29.1 (1.4)	29.2 (1.3)	28.7 (1.5)	0.003
Birth weight, g, mean (*SD*)	1191.2 (191.8)	1201.6 (188.5)	1138.4 (201.1)	0.016
Male, *n* (%)	209 (53.9)	169 (52.2)	40 (62.5)	0.129
Female, *n* (%)	179 (46.1)	155 (47.8)	24 (37.5)	0.129
Cesarean section, *n* (%)	240 (61.9)	202 (62.3)	38 (59.4)	0.655
Multiple birth, *n* (%)	141 (36.3)	117 (36.1)	24 (37.5)	0.833
SGA, *n* (%)	20 (5.2)	16 (4.9)	4 (6.3)	0.665
IUGR, *n* (%)	18 (4.6)	15 (4.6)	3 (4.7)	0.984
Maternal Age, years, median (IQR)	31 (28,35)	31 (28,35)	31 (29.33)	0.629
Chorioamnionitis, *n* (%)	57 (14.7)	48 (14.8)	9 (14.1)	0.877
GDM, *n* (%)	57 (14.7)	52 (16.0)	5 (7.8)	0.222
Hypertensive disorders of pregnancy, *n* (%)	93 (24.0)	82 (25.3)	11 (17.2)	0.416
Any antenatal steroids, *n* (%)	358 (92.3)	298 (92.0)	60 (93.8)	0.818
Maternal education < high school, *n* (%)	111 (28.6)	93 (28.7)	18 (28.1)	0.603
Intubation, *n* (%)	42 (10.8)	33 (10.2)	9 (14.1)	0.362
Surfactant in delivery room, *n* (%)	226 (58.2)	188 (58.0)	38 (59.4)	0.841
Mechanical ventilation, *n* (%)	74 (19.1)	57 (17.6)	17 (26.6)	0.586
Non-invasive respiratory support, *n* (%)	345 (88.9)	286 (88.3)	59 (92.2)	0.311
Gestational age at discharge, weeks, mean (*SD*)	37.9 (2.4)	37.8 (2.0)	38.6 (3.6)	0.086
Length of stay, days, mean (*SD*)	61.4 (21.8)	60.0 (19.3)	68.1 (30.8)	0.049
Respiratory support, days, median (IQR)	20.0 (6.0, 40.0)	18.0 (6,37)	33.5 (6.3, 57.0)	0.005
Oxygen use, days, median (IQR)	1.0 (0.0, 20.0)	1.0 (0.0,16.0)	5.5 (0.0, 37.5)	0.014
BPD, *n* (%)	62 (16.0)	46 (14.2)	16 (25.0)	0.031

*LRD, late respiratory diseases; SD, standard deviation; SGA, small for gestational age; IUGR, intrauterine growth retardation; IQR, interquartile range; GDM, gestational diabetes mellitus; BPD, bronchopulmonary dysplasia.*

Univariate analyses were performed to identify the factors that could influence LRD. The duration of respiratory support was significantly overrepresented in the group of infants with LRD, compared to those without LRD (*P* = 0.005, Table 1). Infants with LRD were more likely to have a lower GA and BW, longer duration of oxygen support, and longer NICU stay (*P* < 0.05). We also verified that BPD diagnosed at 36 weeks PMA was significantly related with LRD in the univariate analyses (*P* = 0.031). There was a greater mean GA at discharge in the LRD group, but the difference was not significant (38.6 weeks vs. 37.8 weeks, *P* = 0.086). A higher proportion of infants with LRD were male, but the difference was not significant (62.5% vs. 52.2%; *P* = 0.129). None of the other factors were significantly different between the groups.

**FIGURE 2:**
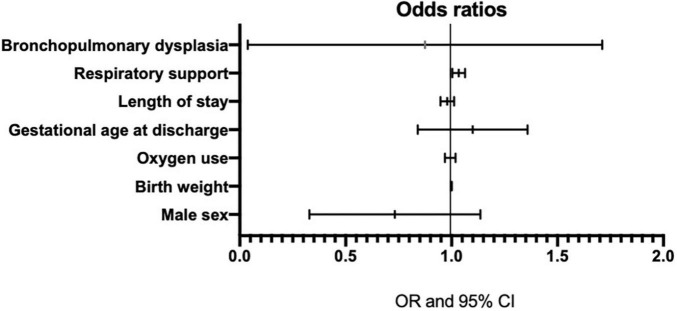
Odds ratios with 95% confidence intervals for model covariates from the logistic regression analysis modeling late respiratory diseases.

Given the number of infants with LRD in our study, seven variables were chosen for multivariable analysis (Table 2). Birth weight was highly correlated with GA. Thus, BW served as an indirect measure of GA when it was included in the multivariable regression analysis. After adjusting for covariates, we found a significant association between the need for respiratory support and LRD at 18–24 months of corrected gestational age (OR: 1.033, 95% CI: 1.004–1.064; *P* = 0.027). With each day of respiratory support, the odds of LRD increased by 1.033-fold. However, none of the remaining factors were significantly associated with the likelihood of LRD. The ORs from the model are graphically ranked in Figure 2.

**TABLE 2:** Parameter estimates from multivariable logistic regression.

Variable	Odds ratio	95% Confidence interval	*P*-value
Male sex	0.658	0.371–1.168	0.153
Birth weight	0.999	0.997–1.001	0.355
Respiratory support	1.033	1.004–1.064	0.027
Oxygen use	0.994	0.969–1.019	0.625
Gestational age at discharge	1.079	0.851–1.368	0.528
Length of stay	0.979	0.948–1.012	0.210
Bronchopulmonary dysplasia	0.608	0.204–1.812	0.372

## Discussion

This retrospective study investigated contributory antenatal and postnatal factors for the development of LRD in preterm infants and found a significant association between respiratory support and the development of LRD by 18–24 months of corrected gestational age. Respiratory support may be an independent risk factor for LRD and could also provide caregivers with practical prognostic information and help researchers identify high risk infants and evaluate future clinical trials for interventions to prevent or mitigate LRD.

In our study, univariate analysis showed that GA, BW, duration of oxygen use, duration of respiratory support, BPD, and NICU stay were associated with LRD. Similar to the results of our study, several other investigations have identified several risk factors for LRD. Morrow et al. (21) reported that BPD, along with other factors, modulates late respiratory outcomes during childhood and reported significant differences in GA, BW, maternal age, maternal smoking, and incidence of chorioamnionitis between patients with and without a LRD diagnosis. The Prematurity and Respiratory Outcomes Program (22), which aimed to identify early predictors of respiratory morbidities, indicated that male sex, ethnicity, intrauterine growth restriction, intubation at birth, maternal smoking, and public insurance could accurately predict LRD. Greenough et al. (14) also reported that four factors—male sex, oxygen dependency at 36 weeks PMA, having older siblings younger than 5 years of age, and living in a rented accommodation were associated with increased respiratory morbidities. These results were not entirely consistent with those of our study, which may be attributed to multiple factors. For instance, study designs and populations were different in each study. In addition, there is no certain definition of LRD, these studies used different methods to evaluate LRD at different time points. Thus, although the results may not be identical, our study nonetheless identified a predictor that may allow us to assess the risk of LRD immediately after preterm birth.

Our results demonstrated that only respiratory support was significantly associated with increased odds of LRD during early childhood. The results were consistent with the study of Jensen et al. (23), which demonstrated that the mode of respiratory support is the best predictor for serious respiratory morbidities. Our results were also in accordance with an earlier study conducted by Isayama et al. (18), which reported that the requirement for oxygen or respiratory support is a better indicator of chronic respiratory insufficiency than oxygen supplement. Mourani et al. (24) found that early pulmonary vascular disease and mechanical ventilation support at 7 days of age are risk factors for LRD. It highlighted the importance of early optimal respiratory managements.

In our study, we found that the LRD rate (16.5%) was relatively lower, whereas Morrow et al. (21) and Keller et al. (22) reported the LRD rates of 69.3 and 68.6%, respectively. Several factors may have contributed to it. Firstly, no uniform definition of LRD has been established. Thus, its operative definition varied across studies. The infants with LRD in our study was relatively severe, this may miss the infants with mild/moderate cases. Secondly, our study population differed from that of other cohort studies, and the higher cutoff for GA may have contributed to the lower incidence of long-term respiratory diseases. Thirdly, it has been documented that an outborn status was a risk factor for severe morbidity in premature infants (25). Given that the infants who participated in our study were inborn and were provided with standard care within the same unit, it may have reduced the incidence of LRD.

As our participants underwent unified management, there were consistent and standardized indications for respiratory support, which adds to the credibility of the difference in our results. This study also had a high follow-up rate (94%), and the follow-up evaluation was relatively objective and straightforward, reducing the risk for bias. However, this was a single-center study, the study population was relatively small, the results may not be generalizable to other centers. The respiratory (e.g., lung function testing, lung clearance index) and cardiovascular (e.g., pulmonary artery pressure) functions were also not measured, making it difficult to assess the sensitivity of the predictors to changes in the patient’s physiologic status over time. Furthermore, we did not pay much attention to asthma and respiratory infection patients without hospitalization, as well as the infants need medications. This may underestimate our results, and which might cause the possibility of selective bias. Neither air pollution nor maternal smoking was considered in our study for the reason of low air pollution in our city and rare case of maternal smoking, the further research would focus on these two important issues. Lastly, the mean GA of our study population was 29 weeks of gestation, which is not the population at the highest risk for LRD. It is uncertain whether our results will remain applicable to infants of lower GA. Thus, further studies with larger sample sizes and focus on the population with the highest risk are warranted.

This study determined that perinatal factors were associated with LRD, allowing earlier identification of high-risk premature infants. In addition, identifying high-risk infants early in the course of the disease may provide a therapeutic window to improve the prognoses of advanced respiratory diseases. This factor could also provide caregivers practical prognostic information to make the use of respiratory support more reasonable.

## Conclusion

In conclusion, this study reported that respiratory support was associated with increased odds of LRD. Thus, it is important to identify safe and effective strategies for preventing dependency on chronic respiratory support. Early identification of high-risk preterm infants may also provide a critical window for applying these established or emerging therapies to improve late respiratory outcomes.

## Data Availability Statement

The original contributions presented in the study are included in the article/supplementary material, further inquiries can be directed to the corresponding author.

## Author Contributions

LS was involved in conceptualization, acquisition of data, preliminary data analysis, outcome assessment, and writing of the original draft. YB was involved in conceptualization, data analysis, and writing of the draft. HZ was involved in acquisition of data, outcome assessment, and writing of the draft. JZ was involved in supervision of the design and execution of the study, final data analysis, and writing of the manuscript. All authors read and approved the final manuscript.

## Conflict of Interest

The authors declare that the research was conducted in the absence of any commercial or financial relationships that could be construed as a potential conflict of interest.

## Publisher’s Note

All claims expressed in this article are solely those of the authors and do not necessarily represent those of their affiliated organizations, or those of the publisher, the editors and the reviewers. Any product that may be evaluated in this article, or claim that may be made by its manufacturer, is not guaranteed or endorsed by the publisher.
